# Patient experience of imaging reports: A systematic literature review

**DOI:** 10.1177/1742271X221140024

**Published:** 2023-01-27

**Authors:** Charlie Rogers, Sophie Willis, Steven Gillard, Jane Chudleigh

**Affiliations:** 1 King’s College London, London, UK; 2 Health Education England, Cambridge, UK; 3 City, University of London, London, UK

**Keywords:** Mixed-methods systematic review, qualitative, radiology, thematic synthesis

## Abstract

**Introduction:**

Written reports are often the sole form of communication from diagnostic imaging. Reports are increasingly being accessed by patients through electronic records. Experiencing medical terminology can be confusing and lead to miscommunication, a decrease in involvement and increased anxiety for patients.

**Methods:**

This systematic review was designed to include predefined study selection criteria and was registered prospectively on PROSPERO (CRD42020221734). MEDLINE, CINAHL, Academic Search Complete (EBSCOhost), EMBASE, Scopus and EThOS were searched to identify articles meeting the inclusion criteria. Studies were assessed against the Mixed-Methods Appraisal Tool version 2018 for quality. A segregated approach was used to synthesise data. A thematic synthesis of the qualitative data and a narrative review of the quantitative data were performed, and findings of both syntheses were then integrated.

**Findings:**

Twelve articles reporting 13 studies were included. This review found that patients’ experiences of imaging reports included positive and negative aspects. The study identified two main themes encompassing both qualitative and quantitative findings. Patients reported their experiences regarding their understanding of reports and self-management.

**Discussion:**

Patient understanding of imaging reports is multi factorial including medical terminology, communication aids and errors. Self-management through direct access is important to patients. While receiving bad news is a concern, responsibility for accessing this is accepted.

**Conclusion:**

A patient-centred approach to writing imaging reports may help to improve the quality of service, patient experience and wider health outcomes.

## Background

The way in which people engage with health care is changing. The patient-centred care (PCC) approach has seen a broad increase in research and quality improvement. PCC aims to increase engagement with systems and services, while challenging traditional power dynamics between the provider and the patient. Involving people in their own care transforms a patient’s role from a passive consumer of health care to an active manager of their own care.^1^ There is no consensus on a unified definition for PCC; current models advocate for a system where people are at the centre of their health care decisions, key principles are aligned with clear communication, respect for values and needs is evident, coordination of care is pivotal and care is individualised and people are supported to make healthcare decisions.^2^ Support for PCC approach is widespread and can be seen within global health care communities, and NHS (National Health Service) long-term plan.^3^ However, the difficulties and barriers that providers face when trying to effect real world change in healthcare culture can produce part measures that fall short of PCC goals.^4^

Implementation of electronic patient record systems (EPRS) that share individuals’ personal health records, are often proposed as a PCC-centric method of communicating with patients about their care.^5^ Patients increasingly have access to their records through EPRS, allowing them to manage appointments, prescriptions and access examination reports. This is likely to become an integral part of the way people access their healthcare information.^6^ The General Practitioner Committee (GPC) 5-year deal decided ‘All patients will be able to have digital access to their full records from 2020’^7^ in England. The COVID-19 pandemic pushed back the deadline for accelerated access to records until summer 2022.^8^ Simply sharing all health information with patients without considering how the experience of navigating personal medical information can facilitate improved health outcomes may lead to patients being overwhelmed by complicated medical terminology and negatively impacting their health.^9^

General health information is considered ‘too complex’ for over 60% of adults, with those most negatively affected being from minority groups and/or on low incomes.^10^ The COVID-19 infodemic, which called for people to find and assess healthcare information at an unprecedented pace, has highlighted how underestimated low health literacy is as a global problem.^11^ Health literacy is defined as a person’s knowledge and competency to find, understand and apply health information to make healthcare choices in their life.^12^ Low health literacy is linked with negative health outcomes, increased use of hospital services and higher levels of mortality in older populations.^13^ It can be conjectured that this is directly applicable to imaging reports, which are typically designed to communicate information between imaging practitioner and referring physician and as such contains complex terms, measurements and subtle caveats. As gatekeepers, health care practitioners have a key role to play in making changes to practice, to remove barriers to patients’ understanding and accessing health information.^1^

The potential influence of removing barriers in relation to imaging reports can be seen when looking at the scale of imaging examinations undertaken. In the 12 months from June 2020 to May 2021, 38.2 million imaging examinations were undertaken in NHS hospitals in England.^14^ This figure shows the massive scope of imaging services that support many NHS pathways and form an integral part of many patients’ care.

Following most imaging examinations, a written report is produced to communicate findings to the referring health care practitioner. Historically, imaging reports were the primary communication between imaging practitioners and referrers, the latter would traditionally be responsible for communicating an interpretation of the report’s relevant findings to the patient. Imaging reports are increasingly being accessed by patients as a part of their electronic record.^9^ However, the imaging report, as a form of stand-alone communication, is vulnerable to error and misinterpretation.^15^ A recent study from Lee and Whitehead found a difference in the perceived meaning of common imaging report terms such as ‘normal’ between radiologists and non-radiology clinicians, showing that while report writers are aiming for clarity there is often misunderstanding for the reader.^16^ A further study from the United States found only 4% of reports sampled were readable at the average adult reading level^17^ with US reading levels ranked similarly as those in the United Kingdom by the Organisation for Economic Co-operation and Development.^18^ This disconnect, between inferred meaning and received understanding between clinicians who share a common medical language, and the level at which imaging reports are written, raises concerns for how patients experience imaging reports.^19^

Sharing imaging reports directly with patients has been suggested to offer benefits. These include the opportunity to identify errors, address any findings deemed ‘clinically non-significant’ by the communicating physician and act as an aide-mémoire to support the patient’s pathway through ongoing care. Due to ongoing NHS shortages in general practice, patients waiting for results to be communicated by a referring physician are experiencing long delays.^20^ The timely sharing of reports directly to patients has been proposed as an initiative to reduce anxiety and subsequent delays in accessing care.^21^ However, concerns have been raised by practitioners regarding patients’ understanding of historical and current radiological reports and the potential for this to contribute to increased anxiety.^22^

A recent literature review examined patient, clinician and radiologist perspectives on direct access to imaging reports. The review by Alarifi et al.^23^ focused on limitations of current radiology information in electronic records and how to improve communication to patients suggesting further work to understand patients’ experiences, needs and concerns through social media. Several studies have explored aspects of how patients experience imaging reports, however, to date, no attempt to synthesise the literature around this topic has been made.^24–35^ To better understand how patients experience imaging reports, a systematic review of the literature was undertaken. The objectives of this review were to

1. Understand patients’ experiences of imaging reports;
2. Determine key areas of importance when communicating imaging findings to patients;
3. Use this information to inform further research and influence service delivery.

## Method

This systematic review followed the principles of the Joanna Briggs Institute (JBI) Reviewers Manual.^36^ This review is reported in line with relevant criteria of the Preferred Reporting Items for Systematic Reviews and Meta-Analyses (PRISMA) statements and Enhancing Transparency in Reporting the synthesis of Qualitative Research (ENTREQ) guidelines.^37,38^ A prospective review strategy was registered with PROSPERO in November 2020 (CRD42020221734).

### Inclusion and exclusion criteria

As this review is interested in all patient-reported feedback on experience, both qualitative and quantitative data were included.

Studies that reported patient-experience data of imaging reports were included. Studies that sought patients’ opinions on hypothetical scenarios were excluded. Studies where patient-reported data could not be extracted from clinician- or health care professional–reported data, were also excluded. Studies that reported on patients’ experience of image acquisition were excluded. Conference abstracts were excluded.

### Search strategy

**Figure 1.**
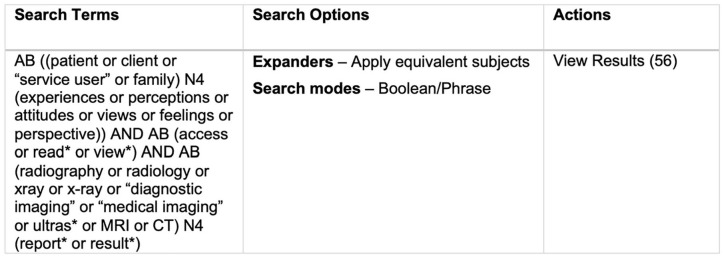
Database search string example.

MEDLINE, CINAHL, Academic Search Complete (EBSCOhost), EMBASE, Scopus and EThOS were searched to identify all published literature in September 2021. An example of a search string used can be seen in Figure 1. Reference lists for all relevant papers were hand-searched to identify further studies. Searches used free-text terms relating to (1) patients, carers and family; (2) self-reported experience feedback; (3) all imaging modalities of interest; (4) reports or results. Searches were re-run in November 2021 to ensure no further relevant papers had been published. No additional papers were identified.

### Screening

Titles and abstracts for each paper were assessed against inclusion/exclusion criteria by two reviewers (C.R. and J.C.). Full text was obtained for all potentially relevant articles and were independently reviewed by two researchers (C.R. and J.C.), to assess eligibility in a standardised blinded manner. Disagreements between reviewers were discussed with a third reviewer (S.W.) and resolved by consensus.

### Data extraction

The JBI mixed-methods data extraction tool was adapted for data extraction of qualitative studies in this review. The following information was extracted from each study: (1) methodology; (2) participants and characteristics; (3) phenomena of interest; (4) setting; (v) outcomes – for qualitative studies, all text related to the review objectives within results, analysis, discussion or appendices were collected; (6) authors conclusion; (7) reviewers’ comments. For studies reporting data from mixed sources, only patient-reported data were extrapolated.

### Risk of bias assessment

All studies were assessed against the Mixed-Methods Appraisal Tool (MMAT) version 2018.^39^ The MMAT was piloted for use in this study by two reviewers (C.R. and J.C.) against a 20% sample of studies to ensure consistency and relevance. A second reviewer (C.R.) then assessed the quality of all studies. Disagreements between reviewers would have been discussed with a third reviewer (S.W.) and resolved by consensus, however, none arose. No studies were excluded based on quality assessment, to avoid valuable insights being omitted.

### Analysis

This review adopted a segregated approach where the qualitative and quantitative evidence was synthesised separately. This method involved two stages; first, a thematic synthesis of qualitative data was performed followed by a narrative review.

Based on thematic analysis, a widely used method for primary analysis of studies, thematic synthesis is often used to bring together and interrogate findings of studies to address research questions about peoples’ experiences.^40^ Thematic synthesis was selected for this review to enable descriptive themes to be generated that go beyond the content of the selected studies to draw on key messages from a limited range of primary studies with differing research aims.^41^

Thematic synthesis was conducted as described by Thomas and Harden.^40^ This process involved inductive line by line coding of all data collected from the studies based on meaning. Coding was approached iteratively with new codes emerging and developing as each study was added. Codes were explored for consistency and crossover addressed as required. Codes were organised and compared for development into categories which were in turn explored in-depth to develop themes.

Second, a narrative synthesis of the quantitative data was performed, which sought to focus and represent the cumulative findings in the data.^42^ Due to the diverse range of measures, research objectives and participants in the identified studies, a meta-analysis was inappropriate. A narrative synthesis is also desirable for implementation in policy and practice and to add meaning/value to quantitative findings.^43^

## Results

### Study selection

**Figure 2.**
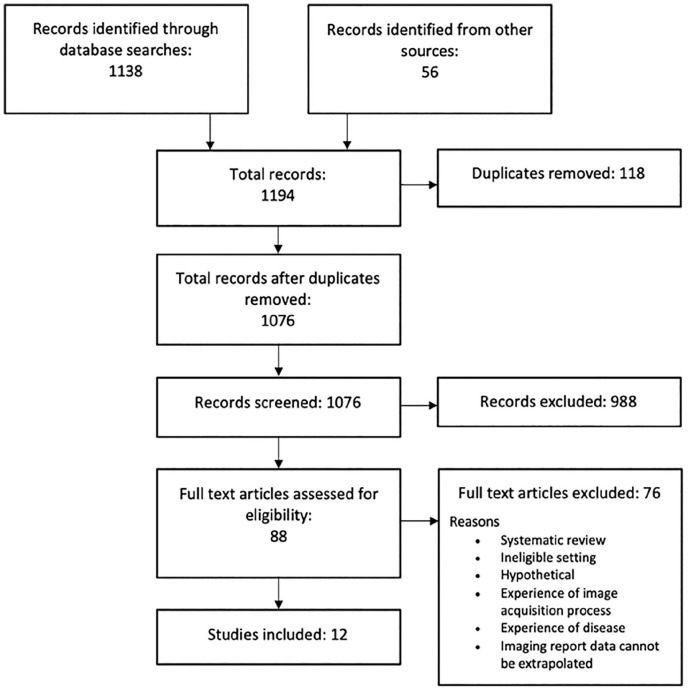
Search strategy.

Of the 1138 records identified through database systematic searches, 988 were removed at the title and abstract screening stage. A further 56 records were included from other sources including grey literature and citation/reference searches. Full text was retrieved for 88 articles. Of these, 76 were excluded due to the reasons identified in Figure 2. During the screening review, two reviewers (C.R. and J.C.) were undecided on the inclusion of one article due to the nature of the measure and if the questions therein qualified as relating to patient experience. The full text article was retrieved and discussed with a third reviewer (S.W.) and a consensus to exclude was reached.

### Study characteristics

Twelve papers (described in Table 1) reporting 13 studies were included in this review.^26,29^ One paper by Hong et al.^30^ reported three studies within one paper; two of these studies fulfilled the selection criteria and were included. Six studies reported qualitative findings,^24,30,31,34,35^ two were ;quantitative.^28,33^ Five studies reported both qualitative and quantitative findings.^25–27,29,32^ Ten studies were conducted in the United States,^26–35^ one in Denmark^25^ and a further two studies were conducted by US researchers online in English-speaking forums.^24,30^ These articles explore patients’ experiences of imaging reports in several settings detailed in Table 1. Two studies^26,29^ incorporated provider/clinician perspectives in their study design, which will be separated out for this study.

**Table 1.** Included study characteristics.

	Study	Study design	Geographic	Research question	No. of participants	Participant characteristics	Setting
**1**	Alarifi et al.^24^	Qualitative	US-based English language online discussion forums	Investigate patient needs and understand information gaps in radiology reports using patient questions posted on online discussion forums	659 questions taken from 4 online forums (Yahoo answers, Reddit, Quora and Wiki answer)	Anonymised forum questions	Internet question and answer discussion forums. Yahoo answers, Reddit.com, Quora and Wiki answer
**2**	Baun et al.^25^	Mixed methods	Odense, Denmark	Investigate the attitudes and experiences of Danish patients with metastatic breast cancer in using electronic health records to view their own scan results	38 survey (four focus groups)	Women; age 42–84 years; 22 undergrad or higher education level (focus group – four women, early 40s to late 70s; two married, and two living alone; one technical training, two undergraduate and one masters)	Single-centre study involving patients with metastatic breast cancer prospectively from January to May 2018 at the Department of Nuclear Medicine, Odense University Hospital, Odense, Denmark
**3**	Bavadian et al.^26^	Mixed methods	United States	Report the feasibility and initial results of a multi-institutional quality improvement project implementing patient and provider feedback for radiology reports (pilot)	367 (219 patients, 148 providers)	Not reported	Patient portal called MyChart and provider portal called Hyperspace. October 2019 to June 2020
**4**	Cook et al.^27^	Mixed methods	United States	Evaluate a web-based interface that presents reports of knee MRI (magnetic resonance imaging) examinations with annotations that include patient-oriented definitions, anatomic illustrations and hyperlinks to additional information	22 survey participants	Not reported	Patients who accessed and reviewed their annotated reports through PORTER’s web-based user interface
**5**	Garry et al.^28^	Quantitative	United States	Comparison of patient satisfaction and understanding of radiology results when received through an electronic patient portal versus direct communication from providers	1005 (486 direct access, 396 referrer communication)	Women 704, men 301; average age 56 (SD 16)	NYU Langone Health, a large urban academic health system, which uses the EHR platform Epic and the patient portal system MyChart
**6**	Henshaw et al.^29^	Mixed methods	United States – Hawaii	Describe patterns of manual release of radiology reports by referring physicians, and the experiences of patients and referring physicians during the first year that the option to release radiology reports was available	508 participants	Not reported	Kaiser Permanente Hawaii, one of seven regions of the nation’s largest not-for-profit integrated health care delivery system
**7**	Hong, 2017 (S1)	Qualitative	US-based English language online discussion forums	Identify patients’ information needs by analysis of questions posted in an online discussion forum	480 comments	Not reported	medhelp.com, helathboard.com, csncancer.org and reddit.com
**8**	Hong et al.^30^	Qualitative	United States	Field study of a prototype to support collaborative review and communication of radiology report content	14 patients and their parents (28 total)	Women 4, men 10; average age 15.4. (parents average age 39.1, 11 women, 3 men)	Children’s Healthcare of Atlanta (CHOA). July 2016 to August 2016
**9**	Johnson et al.^31^	Qualitative	United States	Seek patients’ perspectives on radiology reporting systems, so that reporting systems can begin to be reorganised and made more patient-centered by giving patients greater access to their personal health information	11 participants across 2 focus groups (5 and 6).	Women 9, men 2; 1 Hispanic subject, 5 Black subjects and 5 non- Hispanic White subjects	All outpatients who had recently undergone MRI at a single academic medical centre
**10**	Keselman et al.^32^	Mixed methods	United States	A survey of patients’ experience with reviewing their health records, to identify barriers to optimal record use	104 survey (83 narrative comments)	Women 89, men 14; age: 30–39 – 11; 40–49 – 19; 50–59 – 41; 60 and over – 32; White 95, Asian 2, ‘Other’ 5; ethnicity non-Hispanic 98, Hispanic 2	Online survey posted to 20 high-traffic Google and Yahoo news groups on a range of health-related topics
**11**	Mervak et al.^33^	Quantitative	United States	Identify gaps in practice by analysing priorities patients place on the receipt and comprehension of radiology reports	1489 participants (1597 messages)	Women 543, men 946; White 1323, non-White (African-American 52, Asian 62, Unknown 52)	A large quaternary academic medical centre. October 2014 to December 2014
**12**	Rosenkrantz et al.^34^	Qualitative	United States	Identify factors associated with the patient experience in radiology based on patient feedback reports from a single institution	3675 patient comments	Not reported	A large urban academic medical centre. January 2013 to December 2015
**13**	Woods et al.^35^	Qualitative	United States	Examine patients’ views and experiences with reading their health records, including their clinical notes, online	30 patients (6 family members)	Women 4, men 26 (women 5, men 1); age 49–82	Portland, Oregon VA Medical Center. November 2009 and January 2011

MRI: magnetic resonance imaging; EHR: electronic health record.

### Risk of bias

All studies were assessed using the MMAT, which discourages calculation of a score, instead promoting a more detailed presentation of ratings for each criterion to inform the quality assessment of included studies.^44^ All included studies passed initial screening questions for inclusion in MMAT quality appraisal tool. The MMAT appraisals are detailed in Supplemental Appendix 1 demonstrating how included studies scored against established criterion.

### Qualitative synthesis

The review questions are addressed by two key themes: (1) understanding reports and (2) self-management. Main themes and associated sub-themes are detailed in Table 2. Original data extracts illustrating synthesised findings are provided in Supplemental Appendix 2.

**Table 2.** Main qualitative themes and associated sub-themes.

Theme	Sub-theme
Understanding reports	Overall message of the report
	Medical terminology and language
	Information seeking behaviours
	Comprehension aids
	Errors in reports
Self-management	Direct access
	Aide-memoire for follow-up
	Bad news disclosure

#### Understanding reports

In nine studies, participants struggled to understand their imaging reports.^24–27,29,30,33,34^ The most widely represented theme in this review explores how participants were confused about the overall message that the imaging report was trying to convey. Three studies that solicited open feedback from patients after reading their direct access imaging report indicated that participants were unable to derive an overall meaning from the experience.^26,27,29^ These findings were supported by two studies that collected and analysed online discussion forum content where 26% to 29% of all queries made related to understanding/interpretation of the imaging report.^24,30^ These were a study of Danish patients with metastatic breast cancer^25^ and a further study which interviewed paediatric oncology patients and their parents after using an imaging report comprehension aid.^30^

In addition to being unable to understand overall findings of the imaging report, participants reported specifically struggling with the language and medical terminology used.^24,25,29,30,32,33^ Participants highlighted medical terminology as a barrier to comprehension not only due to their unfamiliarity of the definition of terms in isolation (which could be defined using other tools available such as Internet search engines) but also for a lack of insight into how reporters build these terms together to infer meaning.

After reading their imaging reports some participants demonstrated information-seeking behaviour. Two studies specifically explored instances where participants looked outside of the health care profession for support in understanding their imaging reports.^24,30^ This information-seeking behaviour is seen by participants in three other studies.^26,27,33^ Information seeking behaviours demonstrated included questioning terms and phrasing, asking for a second opinion, seeking meaning for quantitative measurements and reaching out to a health care provider for a face-to-face discussion.

Two of the studies included in this review explored patient experience of using a comprehension aid while viewing their imaging report.^27,30^ Both studies reported mostly positive participant experience with comprehension aids which offered definitions, diagrams and summaries. Cook et al.^27^ reported that 77% of participants found the use of a comprehension aid that helped them to understand their imaging report.

Participants’ ability to understand imaging reports was shown in two studies to be impacted by errors within the reports.^26,34^ Errors included incorrect history, wrong age and typographical error. The inclusion of a participant detectible error affected participant’s confidence in the entire report.

#### Self-management

Five of the studies included in this review reported both positive and negative participant experiences with self-management.^25,29–31,35^ Participants across all five studies reported that having direct access to their imaging report was beneficial for their self-management. Direct access to imaging reports was not only seen as helpful when it was offered but also detrimental when it was not available.^29^ Positive participant experiences included a greater sense of insight and involvement with their own illness, allowing for more shared medical decision-making and detailed communication.^25,30^ Having continued access to imaging reports also allowed participants to review the information before appointments to better inform their discussions, immediately after consultations to solidify meaning^25,30^ and as a follow-up further into their care pathway as an aide-memoire.^30,35^

When exploring self-management in the five studies detailed,^25,29–31,35^ the prospect of participants reading bad news in their imaging reports was explored in only one study, where oncology participants expressed a personal dilemma of whether to read imaging reports alone or to wait until their appointment.^25^ It is important to note, however, that all participants expressed a preference to make the decision themselves and did not want to be ‘protected’ by health care professionals.

### Quantitative synthesis

Data from quantitative studies were organised into two themes: (1) understanding reports and (2) self-management. Main themes and associated sub-themes are detailed in Table 3.

**Table 3.** Main quantitative themes and associated sub-themes.

Theme	Sub-theme
Understanding reports	Understanding the report
	Medical terminology and language
	Information seeking behaviours
	Comprehension aids
	Errors in reports
Self-management	Direct access
	Timing of report
	Bad news disclosure

#### Understanding reports

Three studies presented patient reported experience of understanding imaging reports.^27,28,32^ These studies showed that many patients did not understand their imaging reports, but where available, embedded comprehension aids helped. Cook et al.^27^ found that 82% (*n* = 18) embedded definitions of key works within their imaging reports were easy to understand, and 77% (*n* = 17) found that they helped them to understand their report, with a small amount, 14% (*n* = 3), of participants finding pop-up definitions distracting. Most participants 91% (*n* = 20) also found embedded pictures and drawings to be helpful. Garry et al.^28^ found that 36% (*n* = 320) of participants ‘very clearly’ understood their reports. Further analysis of these data showed that 48% (*n* = 189) of participants who received direct communication from their referrer reported ‘very clear’ understanding in comparison with 27% (*n* = 129) who initially received their report directly. Differences were also noted between imaging modalities with 29% (*n* = 290) of participants reporting a clear understanding of computed tomography (CT) and magnetic resonance imaging (MRI), 38% (*n* = 259) for X-ray and 40% (*n* = 206) for ultrasound. When asked to report ease of comprehension of imaging reports, Keselman et al.^32^ found that 45% of participants indicated that they were easy to understand, 28% gave a neutral response and 27% found it difficult to understand their imaging reports.

Three studies presented patient-reported experience of medical terminology as a barrier to understanding.^25,32,33^ Baun et al.^25^ found that 35% (*n* = 12) of participants viewed access to reports negatively due to the potential risk of reading bad news or of misinterpreting their results. Keselman et al.^32^ found that participants identified the most notable specific comprehension barriers as professional terms, abbreviations and difficult concepts. Mervak et al.^33^ found that out of 1597 patient messages in radiology, 11% (*n* = 168) were specifically asking for clarification of medical jargon.

Three studies presented patient-reported experience of information seeking.^26,29,33^ These studies reported the ways in which participants sought out more information after reading their imaging reports to increase understanding. Bavadian et al.^26^ found that 7% (*n* = 11) of participants requested patient summaries. Henshaw et al.^29^ found that 56% (*n* = 286) of participants did not require any follow-up after reading their imaging reports and 25% (*n* = 126) of participants reported contacting their referrer about their imaging report. Mervak et al.^33^ found a range of information-seeking behaviour from participants. The most frequent request from 12% (*n* = 191) of participants was regarding what the next step in their care pathway would be. Requests for a copy of report/images was made by 6% (*n* = 90) participants. A second opinion on imaging was requested by 4% (*n* = 62) of participants.

Two studies presented patient-reported experience of errors in reports.^26,33^ These studies detailed general and specific errors reported by participants. Bavadian et al.^26^ found 8% (*n* = 13) participants reported errors in their reports. Some examples given include reporting a normal appendix after appendectomy, wrong age and wrong site of pain. Mervak et al.^33^ found that overall, 1% (*n* = 13) of participant messages regarded an error in their imaging report. Types of error are further broken down with typographical errors reported by 0.2% (*n* = 3), the wrong side detailed in 0.1% (*n* = 1) and other perceived errors reported by 0.6% (*n* = 9) of participants.

#### Self-management

Four studies presented patient-reported experience of direct access to their imaging reports.^25–27,29^ These studies showed that most participants found online access easy and thought it was an advantage to be able to directly access their imaging reports. Baun et al.^25^ found that 61% (*n* = 22) of participants thought it was an advantage to see their reports and 44% (*n* = 16) felt that it offered them more insight and involvement with their illness. Bavadian et al.^26^ found that 6% of participants (*n* = 9) responded with a free text response regarding access to reports. Cook et al.^27^ found that 95% (*n* = 21) of participants regarded reading their imaging reports online as helpful. Henshaw et al.^29^ found that 74% (*n* = 377) of participants found direct access to reports easy with an online portal. In addition, 88% (*n* = 446) stated that being able to access imaging reports directly was important to them.

Three studies presented patient-reported experience of the timing of imaging reports.^26,28,33^ These studies showed that many participants were happy with the timing of reports. Conversely, some participants sought out results before they were released to them. Garry et al.^28^ found that 84% (*n* = 840) of participants were either satisfied or indifferent with the timing of their imaging report. Bavadian et al.^26^ found 11% (*n* = 17) of participants responded with constructive text feedback on timeliness of their imaging report. Mervak et al.^33^ found that the most common request through the patient messaging system, from 33% (*n* = 521) of participants, was to chase up their imaging report before it was released to them.

One study presented patient-reported experience of consequences of bad news.^25^ Baun et al.^25^ found that 35% (*n* = 12) of participants felt it was a disadvantage having direct access to imaging reports due to the risk of receiving bad news or the potential for misinterpreting findings.

## Limitations

Eleven of the included studies were US based, which may not accurately represent experiences in the United Kingdom. Two studies collected data via online forums^23,30^ and further five studies did not report any participant characteristics,^26,27,29,30,34^ which impedes any assessment of bias.

The included studies focused their methods on research questions not aligned with the aims of this review, this is a recognised challenge of thematic synthesis.^41^ Differences in how each study interpreted collected data dependent on epistemological approach could impact the translation of concepts across studies, this was minimised by the implementation of a suitable thematic synthesis framework.^40^ The misalignment of aims between primary studies and synthesis also impacted contributions from each primary study once relevant data were extracted.

## Discussion

This review sought to synthesise, for the first time, current evidence on patient experience of imaging reports considering current changes to how a patient can access their current and historical medical records. During the search process, 13 studies, from 12 published papers, containing data representative of patients’ experiences of imaging reports were considered suitable for inclusion. The number of studies is considered disproportionately low considering the search included all types of imaging from all English-writing authors. Ten of the included studies were conducted in the United States and a further two by US researchers in online forums with the final included study based in Denmark. It is not surprising that the United States dominates research regarding patient access to imaging reports as this has been a part of healthcare practice in the United States for some time due to differences in healthcare funding. While the findings herein are relatable to healthcare in the United Kingdom, much more needs to be done to understand the impact of experiencing imaging reports if a quality NHS service is to be offered and harm to patients minimised.

Two main themes were evident encompassing qualitative and quantitative syntheses related to (1) understanding reports and (2) self-management.

Patients understanding of imaging reports can be defined both in terms of their understanding of language used in reports and the overall meaning received from the report. This understanding is impacted by several factors including imaging modality, medical terminology, access to communication aids, and errors in reports.

### Information-seeking behaviours

To improve understanding of imaging reports, patients will display information-seeking behaviours, including reaching out to their healthcare provider for more information, further testing and beyond healthcare professions for support. Doctors, through their professional body (BMA), have expressed how concerned they are about the impact on their limited resources from increased information-seeking behaviours.^45^ Conversely, a UK-based 2014 study showed an 11% reduction in appointments and telephone calls to primary care when patients utilised an electronic health record (EHR) system.^46^ Seeking support outside of healthcare professions can offer insight, expand knowledge and develop a more balanced clinician–patient relationship. However, due to lack of regulation, sheer volume of respondents and vast amounts (90%) of statements contrary to latest medical research,^47^ there is a potential for increased misunderstandings and confusion leading to anxiety, over medication and a deteriorating, strained clinician–patient relationship.^48^

### Errors

This review found that errors in imaging reports have a negative impact on patients’ understanding, not only of the aspect in error but the whole report. Recently a large US study showed that over 21% of participants who read their records through direct access found errors.^49^ Some errors are rapidly resolved to the satisfaction of the patient. However, in some instances, patients face difficulties in having errors corrected leading to emotional and/or psychological distress and delayed diagnosis/treatment.^49^ Direct access offers an invaluable opportunity for patients to identify errors that would, otherwise, go unseen and minimise harm. But, a lack of meaningful change when errors are reported, can impact negatively on clinician–patient relationships and health outcomes.^50^

### Direct access

Patients found accessing their imaging reports directly was easy, but the experience elicited both positive and negative accounts of self-management. A greater sense of insight and involvement is a benefit that is not limited to patients accessing their imaging reports as shown in a recent systematic review of the impact of patient access to their whole electronic record.^6^ Further benefits of self-management from direct access include a decrease in missed appointments, which is a massive problem for imaging departments.^46^

### Unexpected and bad news

The possibility of receiving unexpected/bad news when experiencing imaging reports is a concern for patients, but one they wish to take responsibility for.^25^ Only one of the studies included in this review gave direct access to unexpected/bad news prior to face-to-face discussion.^25^ Another study^30^ implemented a failsafe to ensure that unexpected/bad news was not directly available prior to a discussion with a healthcare professional. Considering the narrow body of this research and the potential for harm, further research into how patients experience communication of unexpected/bad news in imaging reports is needed.

## Summary

This review aimed to (1) understand patient experience of imaging reports, (2) determine key areas of importance when communicating imaging findings to patients and (3) use this information to inform research and influence service delivery. The body of research is narrow, and studies included a focus on elements of patient experience specific to their research aims rather than their whole story. None of the included studies implemented any patient and public involvement (PPI), which would enhance the quality and appropriateness of the work.^2^ This lack of user-focused design limits the scope of this review by omission but does not detract from the findings presented.

## Recommendations

These recommendations for practice are derived directly from the mixed-methods synthesis. Medical terminology should be avoided where possible. Errors in reports, regardless of their impact on diagnosis/summary, should be avoided as they affect the readers’ confidence in the whole report. Where suitable, the use of comprehension aids should be considered.

Future research/service improvements are needed which aim to explore patient experience of imaging reports with appropriate PPI in combination with healthcare practitioners who write imaging reports, to develop interventions that will improve the quality of imaging reports, which address the needs and expectations of both groups.

## Supplemental Material

sj-docx-1-ult-10.1177_1742271X221140024 – Supplemental material for Patient experience of imaging reports: A systematic literature review Click here for additional data file. Supplemental material, sj-docx-1-ult-10.1177_1742271X221140024 for Patient experience of imaging reports: A systematic literature review by Charlie Rogers, Sophie Willis, Steven Gillard and Jane Chudleigh in Ultrasound
